# Pregnancy and medicines: time for paradigm change

**DOI:** 10.1002/jia2.25906

**Published:** 2022-07-19

**Authors:** Agnes Saint‐Raymond, Lynne M. Mofenson

**Affiliations:** ^1^ Paris France; ^2^ Research Department Elizabeth Glaser Pediatric AIDS Foundation Washington DC USA

**Keywords:** pregnancy, public health, drug development, drug safety, drug regulation, clinical trials

1

There is a persistent scarcity of data to support clinical decision making regarding approval and use of drugs, biologics and vaccines in pregnant people, putting them at risk of inadequate, inappropriate or unsafe therapy, which can result in significant health consequences for the mother and her child. In recent years, a number of governmental and non‐governmental organizations have begun to advocate for change in the existing paradigm of medicines development to ensure that women and their health providers can make informed choices about the treatment they need, based on data [1, 2, 3, 4, 5]. The World Health Organization (WHO) and International Maternal Pediatric Adolescent AIDS Clinical Trials (IMPAACT) Network convened a Workshop on approaches to enhance investigations of new drugs in pregnant women in May 2020, which included discussions on the regulatory framework [6]. Building on the outcomes of the discussion, we propose how to leverage the existing regulatory framework and introduce innovations to support earlier investigation of drugs in pregnant and breastfeeding women.

Historically, experience with the teratogenic effects of thalidomide and diethylstilbestrol has led to legal frameworks and regulatory guidance on clinical studies during pregnancy that are risk‐based only, rather than benefit risk‐based, leading to the exclusion of females in medicine development and the lack of robust and reliable data in pregnancy [7, 8]. Additionally, concerns related to any possibility of teratogenicity (even in the absence of preclinical data suggesting such potential) engender liability concerns by industry. As described in other papers in this supplement, as a result, females of childbearing potential are under‐represented and pregnant women excluded from registrational drug trials [6]. Without adequate data on dose and safety, regulators are unable to confidently ensure adequate information about pregnancy in medicine labelling, placing women and healthcare providers in the untenable position of making healthcare decisions in an information vacuum.

In the current regulatory approach, non‐clinical animal data (developmental and reproductive toxicity [DART] studies) required for enrolment of pregnant women are generally not completed until phase III studies in men and non‐pregnant females are already well underway [6, 7, 8, 9, 10]. There have been several recent efforts to close these knowledge gaps and reduce delays from multiple stakeholders with similar conclusions. For example, the PHASES Project “Ending the Evidence Gap for Pregnant Women Around HIV and Co‐Infections” noted three critical conceptual shifts to facilitate the inclusion of pregnant women in research: considering pregnant women as a complex rather than “vulnerable” population; moving from protecting “from,” to protecting “through” research; and promoting fair inclusion rather than presumptive exclusion of pregnant women from clinical drug trials [2]. In a meeting related to vaccines for emerging infectious diseases, the Coalition for Epidemic Preparedness Innovations noted the large gaps and delays in information regarding safety and efficacy of vaccines in pregnant women, advocating for the assessment of vaccine platforms for suitability of maternal immunization early in vaccine development and inclusion of studies in pregnant women in vaccine trials for pathogens that affect women of childbearing potential [11]. Other examples include the US Task Force on Research Specific to Pregnant and Lactating Women [1] and initiatives at the European Medicines Agency, the United Kingdom Medicines and Healthcare Products Regulatory Agency, and other regulatory authorities in the International Coalition of Medicines Regulatory Authorities.

It is time to act. A number of the recommendations that follow in this paper were included in a recent “Call to Action” issued in December 2021 by WHO, IMPAACT and the International AIDS Society [5]. One method regulators could use to change the paradigm and facilitate the inclusion of pregnant people in clinical trials is to take a similar route as has been taken to ensure the evaluation of drugs in children. In Europe, the European Medicines Agency requires “paediatric investigation plans” to be submitted, discussed and agreed by pharmaceutical companies around the time phase II studies are being conducted, and the US Food and Drug Administration either provides incentives to companies if they voluntarily conduct paediatric studies (Best Pharmaceuticals for Children Act) or requires companies to assess medicines safety and effectiveness in paediatric patients with submission of a paediatric study plan (Pediatric Research Equity Act) [12, 13]. These plans are discussed among multiple regulators to avoid regional and national inconsistencies.

Regulators should consider requesting from sponsors during drug development “maternal investigation plans” (MIP) for medicines needed by pregnant women or those of childbearing potential, with careful consideration of the balance of benefits and not just the risks. This means considering the benefit of treating the disease versus the risk of leaving it untreated or treated suboptimally and working with stakeholders to agree on development priorities for new products, as well as for marketed medicines for which we are still missing crucial data. The MIP should include a description of the overall medicine development with timelines; plans for refinement and earlier completion of DART studies; use of modelling and simulation; systematic collection of pharmacokinetics and pregnancy‐specific safety outcomes pre‐licensure; and include a plan for active safety surveillance post‐marketing. Development of a “global” standardized MIP used by regulators in different countries could facilitate pharmaceutical company submissions. Inclusion of pregnant women in phase III trials prior to approval would have to be discussed at predefined timepoints during drug development. The default position would be changed from “presumption of exclusion” to “presumption of inclusion” of pregnant women, with exclusion requiring justification on specific (as opposed to generic) grounds. With or without financial incentives, eventually, this would need to be mandated as part of the legal requirements of the MIP, but such a systematic engagement of regulators with developers can be initiated without waiting for legislative changes.

As an interim step towards requiring an MIP, regulatory agencies can encourage pharmaceutical company implementation of the key principles outlined in the Call to Action and described in this supplement [5, 6]. Regulators should work with relevant stakeholders, including the community of people living with HIV, to agree on development priorities for new products, as well as for marketed medicines for which we are still missing crucial data.

Post‐approval efficacy and safety studies also need to be proactively planned, to collect data on pregnancy outcomes of exposed women in sufficient numbers to be able to have confidence in efficacy and safety data. Regulators can promote and support the use of standardized, harmonized definitions and methods for active surveillance of safety of medicines in pregnancy; for example, the MIP could include a requirement of pharmaceutical company support for the Antiretroviral Pregnancy Registry [14]. Regulatory guidance on medicine use in pregnancy should be coordinated across countries and organizations to avoid conflicting messages. Finally, what is proposed here applies to, but not just to people living with HIV.

## COMPETING INTERESTS

The authors have no competing interests.

## AUTHORS’ CONTRIBUTIONS

All authors contributed equally to the text.

## FUNDING

No funding was received for this article.

## DISCLAIMER

The positions expressed in this article are personal views of the authors and may not be understood or quoted as being made on behalf of WHO, EMA, or any other organization or one of their Committees.
